# Power Surge: Renewed Interest in Nuclear Energy

**DOI:** 10.1289/ehp.113-a742

**Published:** 2005-11

**Authors:** W. Conard Holton

Just past its 50th birthday, commercial nuclear energy is experiencing a tentative rejuvenation that could result in a greater role as a global source of electricity. Skeptics still harbor many of the objections that have slowed or stopped the construction of new nuclear power plants, but rising concerns about the cost and security of energy supplies and global climate change have reframed the debate in terms more favorable for nuclear power advocates.

As a result, the question of whether governments should encourage the construction of new nuclear power plants is no longer off the table in developed countries such as Australia, the United Kingdom, and the United States. For other developed countries such as France and Japan, and for countries with fast-growing economies such as China and India, nuclear energy has remained a central component of energy policy. For example, to achieve its goal of generating 4% of electricity from nuclear power, China plans to add more than 30 new nuclear plants by 2020 to the 11 currently in operation or under construction. India’s goal is to supply 25% of its electricity from nuclear power by 2050.

Worldwide there are now 440 nuclear power reactors operating in 31 countries and producing a combined capacity of 367 gigawatts electric, or about 16% of the world’s supply of electricity. The Vienna-based International Atomic Energy Agency (IAEA)—the agency of the United Nations chartered to promote cooperation on nuclear issues—estimates that at least 60 new nuclear plants will be constructed in the next 15 years. Given the world’s growing demand for electricity, however, this added capacity will still account for only 17% of global electricity use.

## Environmental Conundrum

One central issue facing policy makers and electric utilities is the question of how to meet the rapidly growing worldwide demand for electricity while not increasing global greenhouse gas emissions. The U.S. Department of Energy’s Energy Information Administration tracks world energy trends and projects a 75% increase in global electricity use between 2000 and 2020. By 2050 a tripling of use is probable. Electricity production currently is responsible for an estimated one-third of all greenhouse gas emissions.

In terms of human welfare, this growth in electricity usage is desirable as reflected in the strong correlation between electricity consumption per capita and the United Nations’ human development index, which combines indicators of health, education, and economic prosperity. Overall energy consumption per capita in the developing world is less than one-fifth that in the developed world, and as developing countries industrialize, they will tend to seek the least expensive supply to meet their electricity needs. In most cases this means coal-fired plants, which produce significantly more greenhouse gases—primarily carbon dioxide—than other carbon-based sources such as natural gas–fired generators. Nuclear and noncarbon-based renewable sources such as wind and solar power do not directly create greenhouse gases.

Global climate change and the 2005 entry into force of the Kyoto Protocol to the United Nations Framework Convention on Climate Change have spurred new thinking about the potential value of nuclear energy by both environmental groups and the nuclear energy industry. Recently, several prominent environmentalists have publicly supported nuclear energy, including former Anglican bishop Hugh Montefiore, a long-time trustee of Friends of the Earth, and Patrick Moore, cofounder of Greenpeace.

Their support has alienated them from many in their former organizations, but indicates a more nuanced challenge to nuclear energy by some environmental activists, who are perhaps more willing to consider the nuclear option but still do not think it’s the wisest choice. Organizations such as the Natural Resources Defense Council and the Union of Concerned Scientists now talk in terms of the proper role of government in energy policy and ensuring the safe operation of nuclear plants, rather than whether nuclear power should even be considered.

## Minds Differ

The potential for building new nuclear power plants is quite different in different countries. For example, the role of nuclear power is unlikely to change substantially in countries with a flat demand for electricity, such as Japan, which now relies on nuclear power for 30% of its electric capacity and expects to see a population decline, or France, with a stable population and a power industry that is 80% nuclear. On the other hand, the United States, which currently operates 103 nuclear power plants and relies on nuclear energy for 20% of its electricity, expects to see a rising population and consequent greater demand. Developing countries offer the potential for considerably more use of nuclear power, especially as much of their populations will be urban, providing a concentrated market for large electric-generating plants.

So in answer to the question of whether nuclear power makes economic sense, it simply depends—“in some countries it does, in others it does not,” says Alan McDonald, a staff expert in planning and economic studies at the IAEA. “In countries like China and India, you need [every source of power] you can get. Asia has major pollution problems and energy needs. Sometimes it seems to be a matter of national preferences. In countries like Austria and Denmark, nuclear power is anathema; in others like Germany, opinions may be changing. In the United States, Wall Street is very skeptical and will watch developments closely.”

Relative costs of nuclear energy vary depending on what options and factors are being considered, but in general, McDonald says, the up-front costs of nuclear energy are very high while the cost of operation is relatively low. Thus, countries with government-owned electric utilities have an advantage in new power plant construction because they can fund investments more easily than investor-owned utilities, which are subject to the capital markets and the demand for rapid returns on investments.

“Until the Kyoto Protocol, the environmental value of nuclear energy could not be translated into financial terms,” says McDonald. “But now, obtaining greenhouse gas emission permits for a new coal-fired plant in Europe can cost more than the coal itself. Although the United States is not bound by Kyoto, U.S. investors may see the writing on the wall. If the treaty is changed and nuclear power becomes part of the international market mechanism that allows credit for clean energy sources and the trading of carbon emission credits, that would be a big incentive.”

But more nuclear power doesn’t come without potential security threats of another sort. “If the world sees a big increase in nuclear energy, there will be an increased risk of [nuclear arms] proliferation—all things being equal,” McDonald notes. Indeed, the director general of the IAEA, Mohamed ElBaradei, says that recent revelations about undeclared uranium enrichment activities and reprocessing of spent fuel, along with the discovery of an international illicit market in nuclear technologies, underlines the need for improved controls. On 7 October 2005 ElBaradei and the IAEA were awarded the 2005 Nobel Peace Prize for their efforts to stop the spread of nuclear weapons and prevent North Korea and Iran from acquiring nuclear arms.

In response to the threat of proliferation, the IAEA has developed a model Additional Protocol that signatories can add to their IAEA Safeguards Agreements, which address questions of traceability and verification of nuclear materials. The Additional Protocol strengthens safeguards, protects nuclear materials and facilities, and bolsters the systems of nuclear export controls. So far more than 100 countries have added the protocol to their agreements. The IAEA further proposes that future reactor technologies be designed to be more resistant to proliferation, and that the international enrichment and reprocessing of nuclear fuel be centralized in a few countries under a structure that guarantees supply to member nations.

## An Industry with a Storied Past

The question of whether nuclear energy should play a significant role in future electric power generation cannot be separated from its history, the role played by governments, or the nuclear fuel cycle itself. The cycle has always been a focus of concern, from the potential hazards of uranium mining operations, through the processing of uranium into fuel, to the controlled fission process in the reactor core, and finally to the disposal or reprocessing of the fuel and related waste products.

The civilian nuclear power industry was created through U.S. government–electric utility industry cooperation that officially began with the Atomic Energy Act of 1954. Until that point, all U.S. atomic energy resources had been devoted to military activities. President Dwight Eisenhower’s “Atoms for Peace” speech to the United Nations in December 1953 led to the U.S. government’s financial and technical support of commercial nuclear energy. The government also enacted the Price-Anderson Act of 1957, requiring nuclear power operators to carry the maximum insurance offered by private insurance companies but also limiting their liability—a stipulation demanded by the utility companies before they would invest in building nuclear power plants.

The U.S. Navy first developed the now widely used pressurized-water reactor for propulsion in submarines. This design became the basis for the first commercial nuclear plant at Shippingport, Pennsylvania, which began operation in 1957. In the Soviet Union, reactors designed for producing plutonium for weapons were modified and new ones developed to generate heat and electricity. The first such reactor began producing electricity for the city of Obninsk in 1954.

The fostering of nuclear energy was woven into many U.S. foreign policy initiatives during the early days of the Cold War. The United States sponsored the creation of the IAEA as the global manager of nuclear technology and materials, it supported international research reactors and isotopes for nuclear medicine and agriculture, and it helped create a nuclear energy industry in Europe, where coal production was declining and other sources of electric power were limited.

The U.S. commercial nuclear power industry flourished from the mid-1960s through the early 1970s, although the power plants operating then were not economical compared to other sources at the time. Nuclear energy advocates argued that, with moderate and selective government assistance, the technology could cross the economic threshold into widespread acceptance by the utility industry. The U.S. Atomic Energy Commission—which then combined the functions of today’s Nuclear Regulatory Commission (NRC) and Department of Energy—estimated that the United States would exhaust its oil and coal supplies within 100 years and that nuclear energy was the best replacement for fossil fuels in electricity production. The commission optimistically estimated that by 2000 as much as two-thirds of the nation’s electric power could come from nuclear energy.

The peak year for achieving this scenario in the United States was 1973, when 50 orders were placed for new nuclear plants, although in the following years leading up to 1979, cancellations began to exceed new orders. Then, in March 1979, a series of operator errors and miscommunications led to the partial core meltdown in the pressurized-water reactor at Three Mile Island Unit 2. The accident did not result in major damage outside of the core and primary cooling system, and according to all official estimates, the radiation released during the accident was minimal, well below levels that have been associated with health effects from radiation exposure. However, a panicked evacuation of nearby residents took place, followed by extensive investigations and a government-subsidized 10-year cleanup effort. The notoriety of the accident, combined with the high cost of construction, slow regulatory processes, and political opposition, essentially halted the growth of the U.S. nuclear industry. Although numerous nuclear power plants that had been under construction at the time eventually came online, no new U.S. plants were ordered.

The devastating accident at Chernobyl Unit 4 in April 1986 could have been the death knell of the industry worldwide. The steam explosion, fire, and nuclear fuel melting at the site were the result of a flawed reactor design operated by inadequately trained personnel who violated safety procedures. The reactor design widely used for nuclear power in the Soviet Union did not include the containment system used with most Western reactors, and so substantial quantities of radioactive material, dust, and gases escaped into the atmosphere.

The Chernobyl site is now entombed in a concrete structure known as the Sarcophagus, but it is not stable for the long term and is not air-or watertight (a major new Sarcophagus is planned, but funding is slow to materialize). The accident was a deeply traumatic experience for the 350,000 people who relocated from the area. A 30-square-kilometer area around the site remains closed because of high levels of contamination. About 50 people were killed in the initial accident and emergency response. A September 2005 IAEA report, *Chernobyl’s Legacy: Health, Environmental, and Socio-Economic Impacts and Recommendations to the Governments of Belarus, the Russian Federation, and Ukraine*, estimates that around 4,000 people have died or will die as the result of exposure related to the accident. The report observes that “mental health is the largest public health problem created by the accident,” referring to affected residents’ subsequent poverty, substance abuse problems, and “paralyzing fatalism,” manifested as negative self-assessments of health, belief in a shortened life expectancy, lack of initiative, and dependency on assistance from the state.

Even with the resulting public outcry against nuclear power, the world did not halt new construction of nuclear power plants. However, some European countries such as Belgium, Germany, and Sweden began to reconsider their plans for nuclear energy, and eventually developed policies to phase out existing plants. Now some of these countries are under the gun to find replacement energy sources. Sweden, for example, aims to be nuclear-free by 2010, having taken a second reactor offline in June 2005 (the first was closed in 1999). But the remaining 10 plants still supply about half of Sweden’s domestic energy production, according to the World Nuclear Association.

## New/Old Thinking

An influential 2003 report out of the Massachusetts Institute of Technology (MIT), *The Future of Nuclear Power: An Interdisciplinary MIT Study*, spelled out the major areas of concern surrounding nuclear energy and proposed a plan that the authors hoped would allow the United States to resume development of nuclear power in order to reduce greenhouse gas emissions. The study identified the four critical problems that must be overcome for nuclear power to succeed—cost, safety, waste, and proliferation. It also offered policy recommendations for making the nuclear energy option commercially viable, including steps to lower cost and a limited production tax credit to “first movers,” private sector investors who build and then operate new nuclear plants.

“Our recommendations are basically holding up,” says study cochair Ernest Moniz, who is codirector of MIT’s Laboratory for Energy and the Environment and former undersecretary for energy during the Clinton administration. “On the positive side, new regulatory approaches are being developed, the industry’s intent is to build a new reactor, there are more open discussions with environmental groups, and the Energy Policy Act became law,” he says. “On the negative side, the situation with spent fuel management is worse—Yucca Mountain casts a shadow over any decision. And the non-proliferation situation in Iran is a real problem.”

The fate of Nevada’s Yucca Mountain nuclear burial site is unclear. In the face of sustained resistance from the state and citizens groups, the federal government has slowed in its effort to build a long-term geological repository for commercial spent fuel and high-level radioactive waste. Opposition to the Yucca Mountain project is based on a long history of Nevada being a nuclear weapons testing grounds, resentment at becoming a repository for toxic waste generated elsewhere in the country, and concerns that the site is not geologically stable enough to guarantee that the radioactivity will remain confined over the required 10,000-year span. But several more such sites will be needed in future decades if a significant number of new nuclear power plants are built.

Moniz says the MIT study endorses a robust research and development program and tax credits for the nuclear industry. This is because, in the past, there has been considerable regulatory uncertainty, causing prohibitively high financial risk for utility investors. In addition, the true cost of burning carbon-based fuels has not been internalized, meaning that if the health and environmental costs of pollution and greenhouse gases could be factored in, nuclear energy would be very competitive. As a result, public subsidy of noncarbon-based energy sources is justified.

The comprehensive Energy Policy Act of 2005 that Moniz cites provides loan guarantees to develop energy technologies, including nuclear power, that avoid, reduce, or sequester greenhouse gases. It also provides a tax credit of 1.8¢ per kilowatt hour for 6,000 megawatts of capacity at new nuclear power plants (equivalent to the output of about six new plants). Important to the industry, the act provides investment protection against delays in licensing and startup that are beyond the control of industry, including litigation.

The act also provides several billion dollars for nuclear energy research and development, which translates into work on a more cost-efficient and inherently safer generation of reactors known as Generation IV. These reactors achieve greater safety through passive technologies that automatically shut down the reactor in an emergency, bypassing the risk of operator error (humans still control the normal operation and shutdown of these reactors). They are also more efficient and relatively more cost-effective than their Generation III predecessors. In another bow to the environment, the act funds construction of a cogeneration reactor that will produce both electricity and hydrogen, which advocates hope will be a new, carbon-free fuel for automobiles—the single largest source of greenhouse gas emissions.

Finally, the act funds a central nuclear energy program of the Bush administration: Nuclear Power 2010. The program was unveiled in 2002 as a government–industry cost-sharing plan to identify three sites for new nuclear power plants, develop Generation III reactors, and develop a single-license process with the NRC for approval of both plant construction and operation, thereby removing much of the delay and uncertainty for investors.

In response, three consortia of electric utility companies, reactor suppliers, and construction firms have made proposals. None are yet committed to building a new nuclear plant. The consortia are led by Dominion Resources, Exelon and Entergy (via the NuStart Energy Development consortium), and the Tennessee Valley Authority. These consortia represent operators of 67 of the nation’s nuclear plants, and their proposals have all focused on building a new plant on sites where plants already operate—in much the same way that a consortium of 10 electric utilities built the Yankee Rowe plant, one of the first commercial nuclear plants, in the 1950s.

The consortia embrace a number of different reactor vendors and designs, some of which have already been certified by the NRC. The final decision on building a nuclear power plant will depend on factors as they stand later this decade, including the power market, the status of permanent spent fuel storage, and the ability of the participants to obtain financing without adversely affecting their credit ratings.

## Concerned Parties

“The industry’s interest is very real,” says Russ Bell, a senior project manager for new plant development at the Nuclear Energy Institute, a utility trade association. “The utilities are [participating in consortia and spending money on preliminary designs and siting plans] because the economics are turning in favor of nuclear, especially over the long term. [The Kyoto Protocol] is not driving us, but it makes sense and there is increasing concern about pollution in the United States and more stringent environmental regulations.”

Bell says the industry is getting what it needs from the Energy Policy Act and is looking to government to do no more than jumpstart new builds after so much time has passed. He acknowledges the long time horizon for building new plants in the United States. Assuming that any of the consortia meet the 2010 goal of being licensed to build and operate a plant, another four to five years will pass before construction is complete and electricity flows. Meanwhile, the electric utility industry will continue to improve operating performance of existing nuclear power plants and apply for license extensions.

Originally licensed for 40 years, the first operating license issued by the NRC will expire in 2006, approximately 10% will expire by the end of 2010, and more than 40% will expire by 2015. The decision to seek license renewal is strictly voluntary, and nuclear power plant owners must decide whether they are likely to satisfy NRC requirements and whether license renewal is more cost-effective than shutting down and pursuing other sources of energy. The NRC has now granted 35 plants the right to operate for another 20 years. Three-quarters of the nation’s plants have received, have applied for, or are expected to apply for an extension.

The question of plant life extension can bring the relationship between nuclear energy and greenhouse gases into sharp focus. For example, the governors of nine Northeast states have proposed an agreement to cap greenhouse gas emissions from all power plants in their states. Two nuclear power plants in the region, one in Vermont and one in New Jersey, are up for life extension, yet if these plants are shut down, the result would be increased reliance on carbon-based fuels. This could potentially triple greenhouse gas emissions in Vermont and double them in New Jersey, according to the 14 September 2005 edition of *The New York Times*.

“We are not fundamentally opposed to nuclear power,” says David Lochbaum, a nuclear safety engineer at the Union of Concerned Scientists, “but there are better choices. In addition, we now have spent nuclear fuel in storage places where it is not meant to be. It’s not a health threat yet, but it could be.”

Lochbaum is also concerned about the oversight role played by the NRC. “The NRC budget has been cut for a decade,” he notes. “It is understaffed to support a nuclear resurgence. And the industry still has operational troubles at some plants.”

These concerns are echoed by Thomas Cochran, director of the nuclear program at the Natural Resources Defense Council and an advisory committee member on the MIT study. “The Energy Policy Act was the result of successful lobbying by the nuclear industry,” he says. “They will probably build a few plants and then the issue is, are you back to where you are today?” Cochran does not believe that the subsidy or the economics will work for nuclear power. “It’s not helpful to just say you are for or against nuclear,” he says. “Ultimately you must make a decision on real policy to address global warming, and a carbon tax is the best way.”

The objective of a carbon tax would be to internalize the environmental costs and hope for an open competitive market for energy. “To balance the energy market, you either tax a pollutant or regulate it,” says Cochran. “If public policy was made correctly, it would help the nuclear industry.”

Is there a real, economically justified “nuclear resurgence,” or simply a steady growth in some regions to meet rising demand for electricity? Nothing happens quickly in the world of power plant construction. Yet major investments by government and industry can change the bases of electricity supplies in the time frame of a decade or two. France closed its last coal mine in 2004, and its transition from 15% to 80% nuclear-based electricity was accomplished in 20 years. A sense of optimism and urgency now surrounds the question of whether to pursue nuclear power. How this translates into results should unfold at a brisk, measurable pace.

## Figures and Tables

**Figure:**
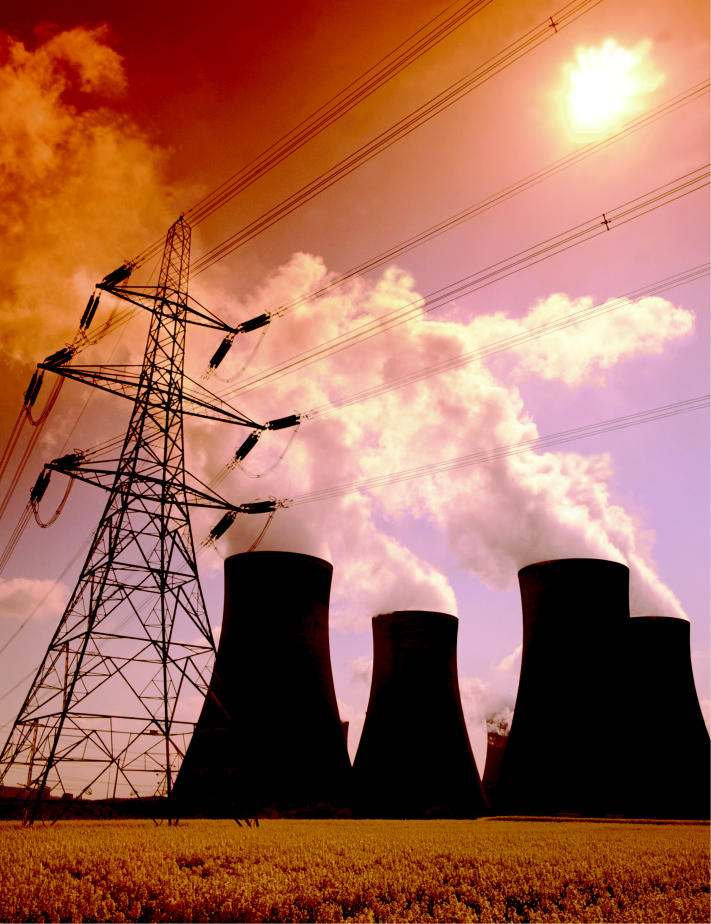


**Figure:**
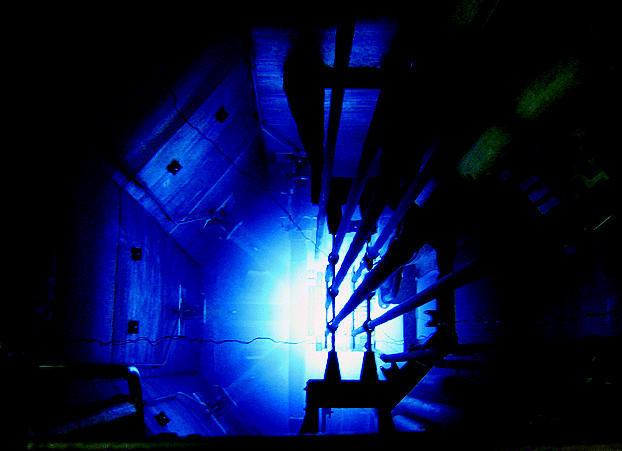
The core of the matter. The view looking down into a research reactor core in Chile shows the fuel elements and control rods hanging in a water pool.

**Figure:**
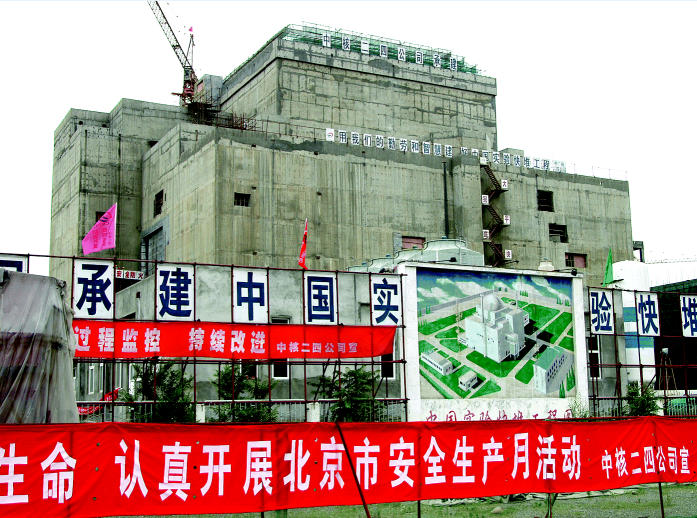
Full steam ahead. Construction is well under way on China’s first experimental fast breeder reactor, located in Tuoli.

**Figure:**
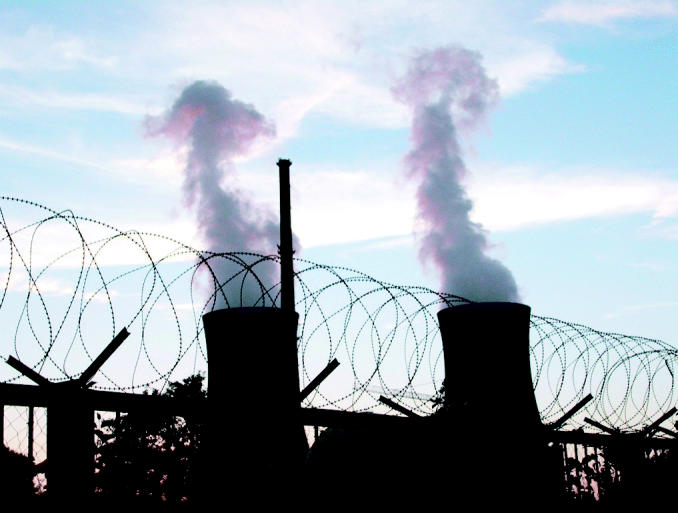
Terror target? Some critics’ reservations about nuclear energy revolve around the fear that reactors and their contents may pose an attractive target for terrorists.

**Figure:**
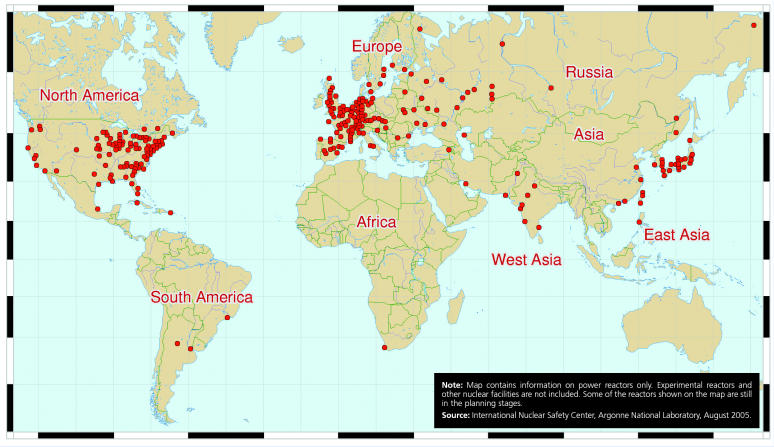
Locations of Nuclear Power Plants Worldwide

**Figure:**
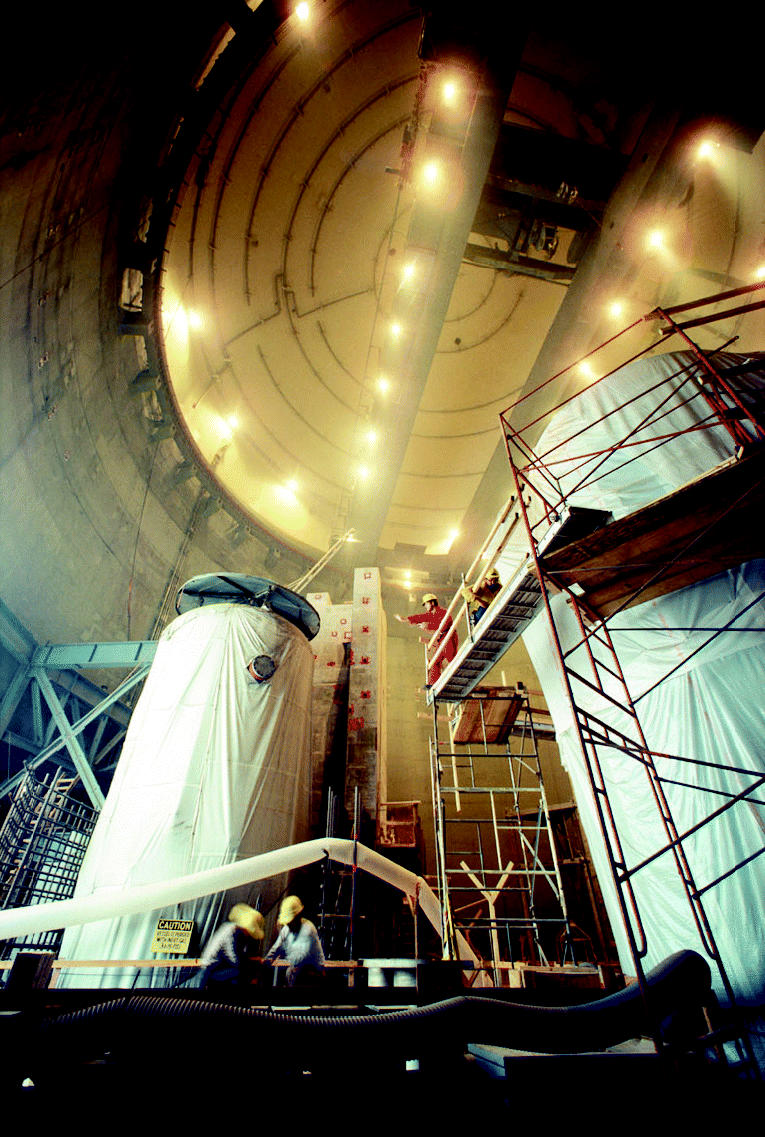
Building on the past. Construction of new nuclear plants continues worldwide. Although stalled in the United States, renewed interest and the need for energy may bring this power source back online.

**Figure:**
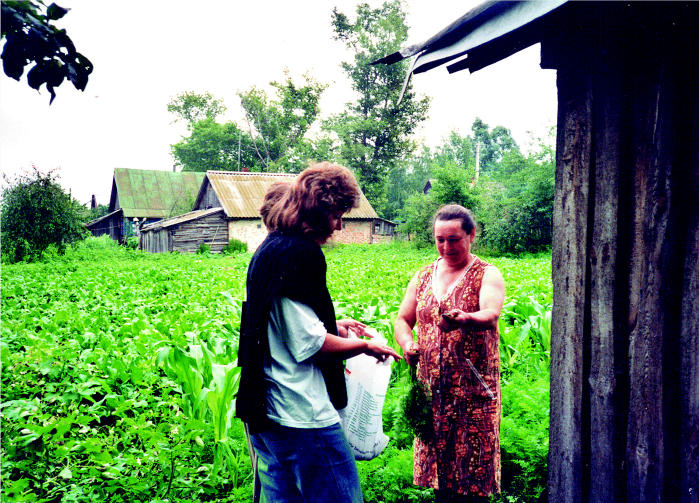
Nuclear fallout. A researcher buys food samples from a local farmer for radionuclide analysis during the International Chernobyl Assessment Project. A recent IAEA report states, though, that the greatest long-term health impact from the accident is psychological trauma.

**Figure:**
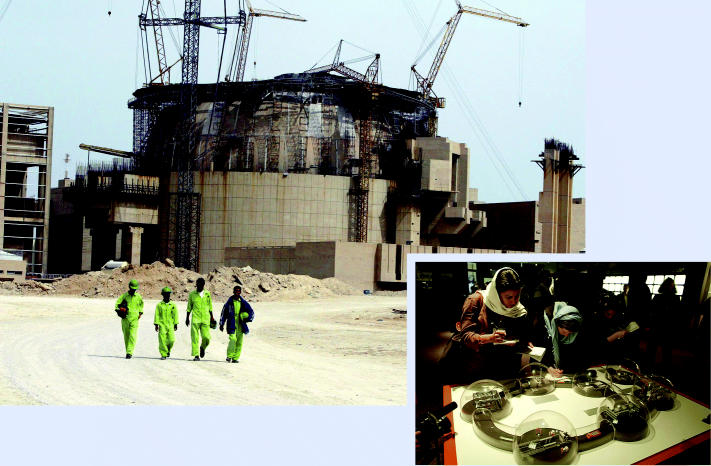
Iran moves ahead. (above) Construction of the Bushehr nuclear power plant, being built under an agreement with Russia, is under way in Iran. (right) Journalists examine a scale model of the Bushehr plant during a visit to the construction site.

**Figure:**
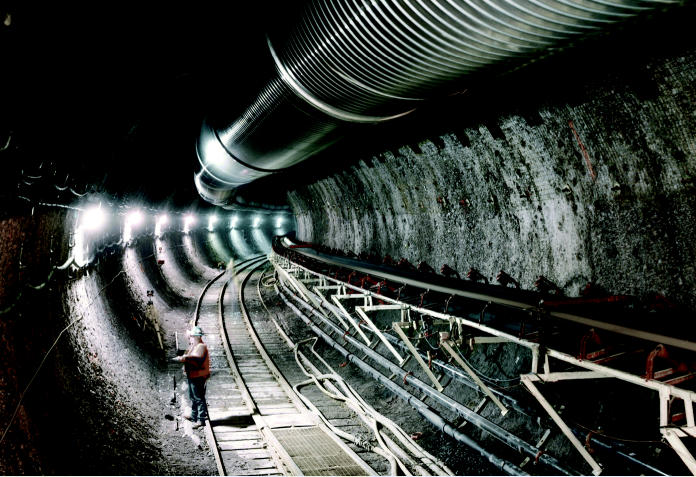
A yucky situation. Yucca Mountain, in the Mojave Desert of Nevada, is the site Congress designated as a geologic repository for the nation’s spent nuclear fuel and high-level radioactive waste. However, the project has been fraught with technical problems and public opposition.

**Figure:**
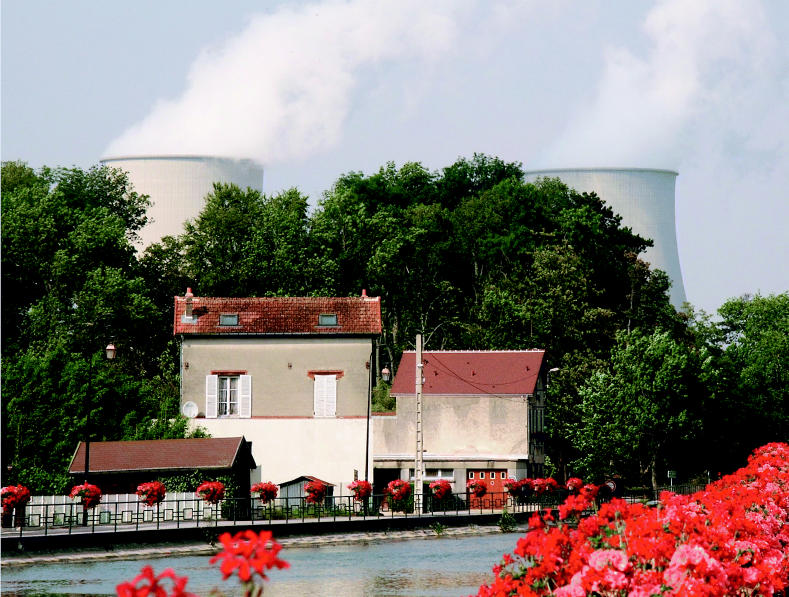
Vive la nuclear! France has embraced nuclear energy and now obtains 80% of its electricity from nuclear power.

**Figure:**
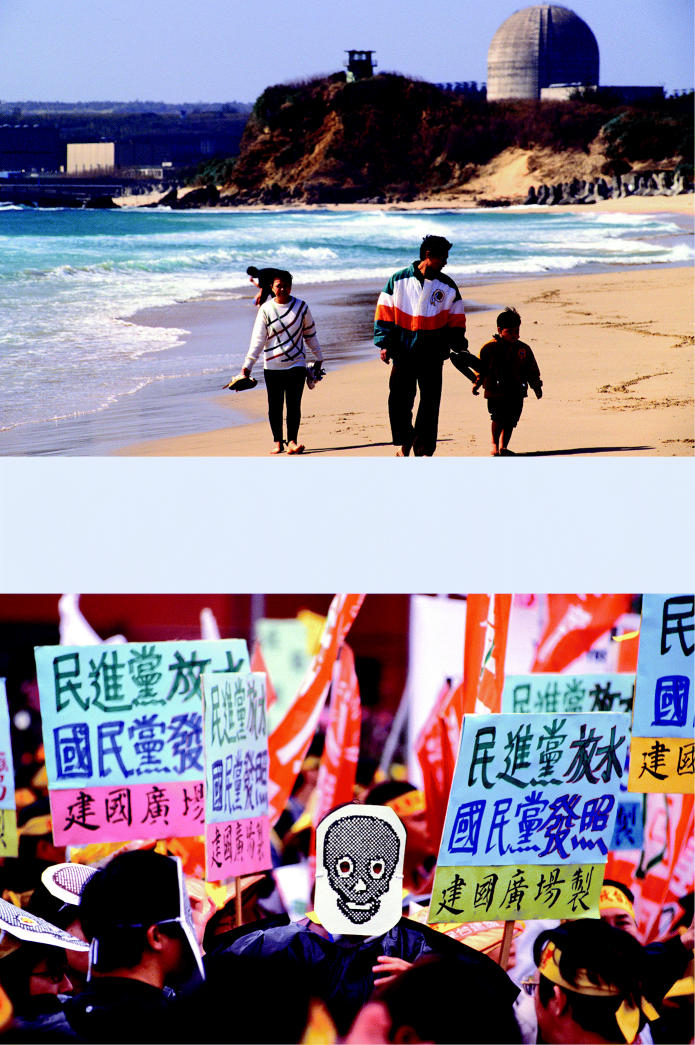
Nuclear microcosm. Many nations have an ambivalent relationship with nuclear energy. (above) A family walks along the beach in Kenting, China, with National Nuclear Power Station No. 3 behind them. Until recently, the waste from this power station was shipped to a controversial storage facility on nearby Orchid (Lanyu) Island. (below) Masked student protestors voice their opposition at an antinuclear rally.

